# Preoperative Overestimation of Disease Extent and Discordance-Driven Mastectomy: A Retrospective Review of 245 Cases

**DOI:** 10.1245/s10434-026-20137-x

**Published:** 2026-07-17

**Authors:** Hadas Kadar Sfarad, Cheng-Bang Chen, Yujie Wang, Mehmet Akcin, Heidi Santa Cruz, Karishma Chapalamadugu, Emily McFarlane, Eli Avisar

**Affiliations:** 1 Division of Surgical Oncology, Dewitt Daughtry Department of Surgery, University of Miami Miller School of Medicine, Miami, FL USA; 2 Department of Industrial and Systems Engineering, University of Miami, Coral Gables, FL USA; 3 Dewitt Daughtry Department of Surgery, University of Miami, Miami, FL USA; 4 Miller School of Medicine, University of Miami, Miami, FL USA

**Keywords:** Breast cancer, Magnetic resonance imaging, Mammography, Imaging–pathology discordance, Mastectomy, Surgical decision-making

## Abstract

**Background:**

Preoperative mammography and magnetic resonance imaging (MRI) guide surgical planning in breast cancer but may misestimate disease extent. We quantified imaging–pathology discordance among patients undergoing mastectomy and estimated the discordance-driven mastectomy rate (DDMR), defined as imaging-extensive disease with localized final pathology, as a measure of discordance.

**Patients and Methods:**

We retrospectively reviewed 245 consecutive mastectomy cases (2018–2019) at a tertiary academic center; 236 had usable postoperative pathology and were analyzed. Preoperative imaging (mammography and/or MRI) was classified as localized versus extensive using predefined extent-indicating features and/or maximum size > 5 cm. Final pathology was similarly classified using size and multifocality/multicentricity as the reference standard. Concordance categories were concordant-localized, concordant-extensive, imaging overestimation, and imaging underestimation. The primary outcome was any discordance; the key clinical outcome was DDMR. Decision drivers were abstracted from the medical record when available.

**Results:**

Overall discordance occurred in 119/236 (50.4%) cases and was predominantly imaging due to overestimation (113/236, 47.9%; 95% CI 41.4–54.5); underestimation was uncommon (6/236, 2.5%). DDMR events occurred only when at least one modality had extent-indicating features and were common with mammography-only (44/53, 83.0%), MRI-only (30/43, 69.8%), or both (39/62, 62.9%). DDMR rates were similar across mastectomy subtypes and by neoadjuvant chemotherapy receipt. Among DDMR cases, surgeon-only requests were most frequent (71/113, 62.8%) versus patient-only requests (31/113, 27.4%; *p* < 0.001). Agreement between imaging and pathology was low (MRI *κ* = 0.241; mammography *κ* = 0.123).

**Conclusions:**

Among a cohort of patients undergoing mastectomy, imaging–pathology discordance was common and predominantly reflected overestimation. These findings highlight the importance of cautious interpretation of imaging-defined extent within clinical decision-making.

**Supplementary Information:**

The online version contains supplementary material available at https://doi.org/10.1245/s10434-026-20137-x.

Accurate assessment of tumor extent is fundamental to preoperative decision-making in breast cancer care. Contemporary breast imaging, principally mammography and magnetic resonance imaging (MRI), plays a central role in defining disease burden, guiding surgical planning, determining eligibility for breast-conserving surgery (BCS), and informing adjuvant treatment strategies.^1,2^ While these modalities have improved cancer detection, particularly in dense breast tissue, preoperative imaging remains an imperfect surrogate for true pathologic extent. Numerous studies have demonstrated meaningful size and extent discordance between radiologic estimates and final surgical pathology, with both overestimation and underestimation observed across imaging platforms.^3–5^ Such discrepancies have direct implications for surgical recommendations and may influence the choice between BCS and mastectomy.

MRI, in particular, is characterized by high sensitivity and relatively lower specificity, a property that enhances lesion detection but increases susceptibility to false positive findings and exaggerated estimates of disease extent.^6–8^ This hypersensitivity may manifest as perceived multifocality, multicentric disease, or extensive ductal carcinoma in situ (DCIS), findings that can shift surgical planning toward mastectomy despite ultimately localized pathology. Prior work has shown that MRI frequently overestimates tumor size compared with histopathologic measurements, particularly in invasive ductal carcinoma, raising concern that imaging findings may contribute to more extensive surgical management.^4,9,10^

Although imaging–pathology discordance is widely recognized, the clinical implications of imaging-defined disease extent for definitive surgical decision-making remain incompletely characterized. Prior population based and institutional studies have demonstrated an association between preoperative MRI use and higher rates of mastectomy among patients who would otherwise be candidates for breast-conserving surgery, without consistent differences in tumor size, margin status, or oncologic outcomes.^1,11,12^ However, these studies have largely focused on imaging utilization rather than directly examining the role of imaging–pathology discordance in driving surgical escalation at the individual patient level. As a result, the extent to which imaging overestimation of disease burden contributes to mastectomy selection, independent of final pathologic extent, remains insufficiently defined.

To address these gaps, the present study examines preoperative imaging–pathology discordance among patients undergoing mastectomy. We examine the frequency and direction of discordance, including cases in which imaging suggests extensive disease but final pathology is localized. Importantly, this study is not designed to determine whether imaging findings directly caused surgical decision-making but rather to characterize discordance patterns within this population.

## Patients and Methods

### Study Design and Patient Selection

We conducted a retrospective review of 245 consecutive patients undergoing mastectomy at a single tertiary academic center between January 2018 and December 2019. Of these, cases missing a usable postoperative reference classification were excluded because imaging–pathology concordance could not be assessed, yielding an analytic sample of 236 patients. Eligible patients were women > 18 years old undergoing mastectomy for invasive carcinoma or ductal carcinoma in situ (DCIS). Cases were excluded if patients had known hereditary cancer syndromes (e.g., BRCA or other germline mutation carriers), inflammatory breast cancer, bilateral disease, locally recurrent cancer, or male breast cancer. Mastectomy procedures included total mastectomy (TM), modified radical mastectomy (MRM), nipple-sparing mastectomy (NSM), and skin-sparing mastectomy (SSM).

This study was approved by the institutional review board (IRB 20053311) with waiver of informed consent.

### Imaging and Pathology Classification

Preoperative imaging consisted of mammography, breast MRI, or both. Digital breast tomosynthesis was included under mammography when applicable. Ultrasound findings were not incorporated into the extent classification framework. Imaging reports were reviewed and abstracted using predefined structured criteria to classify disease as localized or extensive on the basis of modality-specific extent-indicating features.

Preoperative imaging extent was derived from structured abstraction fields capturing modality-specific extent findings. For MRI, the indication variable captured (1) multifocality, (2) multicentricity, (3) extensive non-mass enhancement, (4) satellite lesions, (5) extensive calcifications, or (6) extensive disease; for mammography, the indication variable captured (1) multifocality, (2) multicentricity, (3) satellite lesions, (4) extensive calcifications, or (5) extensive disease. For each imaging modality, findings were classified as extensive if any extent-indicating code was present and/or if the maximum preoperative tumor dimension exceeded 5 cm when a numeric measurement was available. Nonnumeric size entries were treated as missing; in the absence of extent-indicating criteria, imaging was classified as localized. A patient-level preoperative imaging extent classification was then assigned as extensive if either MRI or mammography met criteria for extensive disease, and as localized otherwise. For patients receiving neoadjuvant chemotherapy, imaging classification was based on the most recent preoperative imaging prior to surgery.

Separately, MRI and mammography reports were coded for modality-specific imaging features indicating extensive disease on preoperative imaging (binary per modality) and summarized as neither, MRI-only, mammography-only, or both extensive.

Final postoperative pathology findings were collected for each patient and used as the reference standard for imaging–pathology comparison. Postoperative disease was classified as extensive if the recorded postoperative maximum tumor size exceeded 5 cm and/or if multifocal or multicentric disease was documented; otherwise, it was classified as localized. Nonnumeric or unknown entries in size fields were treated as missing; classification was based on multifocality/multicentricity when available.

Using these two binary classifications, each case in the analytic sample was assigned to one of four predefined concordance categories: (1) concordant–localized: imaging localized, pathology localized; (2) overestimated by imaging: imaging extensive, pathology localized; (3) concordant–extensive: imaging extensive, pathology extensive; and (4) underestimated by imaging: imaging localized, pathology extensive. The primary outcome was imaging–pathology discordance (discordant_any), defined as either overestimation or underestimation. The key clinical outcome was the discordance-driven mastectomy rate (DDMR), defined as imaging overestimation (imaging-extensive disease with localized final pathology).

### Assessment of Clinical Decision Drivers

For each case, the medical record was reviewed to determine whether the decision to proceed with mastectomy was driven primarily by patient preference, surgeon recommendation, both, or neither explicitly documented in charts.

These designations were used to analyze clinical decision patterns within the discordant cohort.

### Mastectomy Subtype Analysis

All concordance analyses were repeated for each mastectomy subtype (TM, MRM, NSM, and SSM), allowing for comparison of discordance and DDMR rates across surgical approaches. Neoadjuvant chemotherapy (NACT) status was recorded and incorporated into subgroup analyses.

### Statistical Analysis

Descriptive statistics were used to summarize patient demographics, tumor characteristics, imaging findings, and mastectomy subtype distributions. Continuous variables are reported as means with standard deviations and were compared using the Wilcoxon rank-sum test. Categorical variables are presented as counts with percentages and were compared using Fisher’s exact test. Analyses were performed using imaging–pathology discordance and discordance-driven mastectomy rate (DDMR) as defined above.

To identify factors associated with DDMR, multivariable regression models were constructed. Because several discordance patterns involved small cell counts and evidence of complete or quasi-complete separation, Firth penalized logistic regression was used to estimate adjusted odds ratios (ORs) with improved small-sample performance.^13^ Covariates included modality-specific imaging features indicating extensive disease, tumor histology, clinical T stage, age, laterality, receipt of neoadjuvant chemotherapy, and maximum preoperative tumor size.

As a sensitivity analysis, Poisson regression (Zou approach) with robust variance was used to estimate risk ratios for predictors of DDMR.^14^ Agreement between imaging and pathology classifications was assessed using Cohen’s *κ* statistic and interpreted using standard benchmarks. A two-sided *p* value < 0.05 was considered statistically significant.

All analyses were performed using R statistical software (version 4.3.2).

## Results

A total of 245 patients undergoing mastectomy met inclusion criteria; 236 had a usable postoperative pathology reference and were included in the imaging–pathology concordance/discordance analyses. Baseline demographic and clinicopathologic characteristics, including age, tumor histology, and receptor status, are summarized in Table 1. Total mastectomy (TM) accounted for the majority of procedures (132/236, 56.0%), followed by modified radical mastectomy (MRM, 38/236, 16.1%), skin-sparing mastectomy (SSM, 36/236, 15.3%), and nipple-sparing mastectomy (NSM, 30/236, 12.7%).

**Table 1:** Patient, tumor, and treatment characteristics stratified by imaging–pathology concordance

Characteristic	Imaging–pathology discordance		Overall *N* = 236^1^	*p* value^2^
	No *N* = 123^1^	Yes *N* = 113^1^		
Age (years)	54.7 (12.2)	55.6 (12.1)	55.1 (12.2)	0.6495
Mastectomy type				0.4308
Total mastectomy (TM)	66 (54%)	66 (58%)	132 (56%)	
Modified radical mastectomy (MRM)	23 (19%)	15 (13%)	38 (16%)	
Nipple-sparing mastectomy (NSM)	13 (11%)	17 (15%)	30 (13%)	
Skin-sparing mastectomy (SSM)	21 (17%)	15 (13%)	36 (15%)	
Neoadjuvant chemotherapy	44 (36%)	41 (36%)	85 (36%)	> 0.9
Family history of breast cancer	38 (32%)	39 (37%)	77 (34%)	0.4830
Preoperative T stage				0.0616
Tis	14 (11%)	8 (7.1%)	22 (9.3%)	
T1	23 (19%)	23 (20%)	46 (19%)	
T2	34 (28%)	36 (32%)	70 (30%)	
T3	28 (23%)	30 (27%)	58 (25%)	
N/A	8 (6.5%)	11 (9.7%)	19 (8.1%)	
Other	0 (0%)	1 (0.9%)	1 (0.4%)	
Preoperative imaging-defined multifocality/multicentricity	12 (9.8%)	1 (0.9%)	13 (5.5%)	
Tumor pathology				0.1693
DCIS-containing	23 (19%)	21 (19%)	44 (19%)	
Invasive ductal carcinoma (IDC)	60 (49%)	48 (42%)	108 (46%)	
Invasive lobular carcinoma (ILC)	16 (13%)	8 (7.1%)	24 (10%)	
Mixed histology	22 (18%)	34 (30%)	56 (24%)	
Others	2 (1.6%)	2 (1.8%)	4 (1.7%)	

^1^Mean (SD); *n* (%)

^2^Wilcoxon rank sum test; Fisher’s exact test

Values are presented as mean (SD) for continuous variables and number (%) for categorical variables. *p*-Values are from Wilcoxon rank-sum tests for continuous variables and Fisher’s exact tests for categorical variables

Using the predefined four-category imaging–pathology concordance framework, cases in the analytic cohort (*n* = 236) were classified according to preoperative imaging extent and final pathologic extent (Table 2). Concordant localized disease (localized imaging and localized pathology) was observed in 72 patients (30.5%), while concordant extensive disease (extensive imaging and extensive pathology) was observed in 45 patients (19.1%). Imaging underestimation (localized imaging with extensive pathology) was uncommon, occurring in six patients (2.5%). Overall, imaging–pathology discordance occurred in 119 of 236 cases (50.4%), of which 113 represented imaging overestimation and 6 represented underestimation, indicating that discordance was predominantly driven by overestimation.

**Table 2:** Imaging–pathology concordance categories and discordance-driven mastectomy rate (DDMR)

Imaging–pathology category	Imaging extent	Pathologic extent	*n*	% of cohort
Concordant–localized	Localized	Localized	**72**	**30.5**
Imaging overestimation (DDMR)	Extensive	Localized	**113**	**47.9**
Concordant extensive	Extensive	Extensive	**45**	**19.1**
Imaging underestimation	Localized	Extensive	**6**	**2.5**
**Total**			**236**	**100**

Values represent the number and proportion of patients within each imaging–pathology concordance category. DDMR is defined as imaging overestimation (imaging-extensive disease with localized final pathology)

Imaging overestimation—defined as imaging-extensive disease with localized final pathology—was identified in 113 patients, representing 47.9% of the analytic cohort (113/236; 95% CI 41.4–54.5). By definition, these cases constituted the discordance-driven mastectomy rate (DDMR) group. Across the analytic cohort, imaging–pathology discordance demonstrated marked directional asymmetry, with overestimation occurring far more frequently than underestimation.

Modality-specific extent-indicating imaging feature flags are distinct from the binary preoperative imaging extent classification used in the concordance framework. Therefore, DDMR was additionally summarized according to imaging-source extent-indicating patterns. No DDMR events were observed among patients with neither MRI-defined nor mammography-defined extent-indicating findings (0/78, 0.0%). In contrast, DDMR was frequent when extent-indicating features were present on mammography alone (44/53, 83.0%), MRI alone (30/43, 69.8%), or both modalities (39/62, 62.9%), indicating that DDMR events were concentrated among cases flagged as extent-indicating by at least one imaging modality.

Across the analytic cohort, preoperative imaging demonstrated substantial heterogeneity in extent-indicating feature attribution: 33.1% of patients exhibited no extent-indicating features on either MRI or mammography (78/236), 26.3% demonstrated extent-indicating features on both modalities (62/236), 22.5% on mammography alone (53/236), and 18.2% on MRI alone (43/236).

Given known differences in imaging performance across tumor histologies, imaging overestimation rates were examined by histologic subtype (Table S1). Mixed histology tumors demonstrated the highest overestimation rate (60.7%; 34/56; 95% CI 46.8–73.2), followed by DCIS-containing tumors (47.7%; 21/44; 95% CI 32.7–63.1), invasive ductal carcinoma (44.4%; 48/108; 95% CI 35.0–54.3), and invasive lobular carcinoma (33.3%; 8/24; 95% CI 16.4–55.3). Across histologic subtypes, discordance remained directionally asymmetric, with overestimation predominating and underestimation remaining uncommon.

DDMR proportions were similar across mastectomy subtypes, with overlapping confidence intervals indicating no clear subtype-specific differentiation. TM demonstrated a DDMR rate of 50.0% (66/132; 95% CI 41.2–58.8), MRM 39.5% (15/38; 95% CI 24.0–56.6), NSM 56.7% (17/30; 95% CI 37.4–74.5), and SSM 41.7% (15/36; 95% CI 25.5–59.2). Rates of DDMR were similar between patients who received neoadjuvant chemotherapy and those who underwent upfront surgery (48.2% [41/85; 95% CI 37.3–59.3] versus 47.7% [72/151; 95% CI 39.5–56.0], respectively; Table S2).

Among DDMR cases with documented decision pathways, surgeon-only requests accounted for 62.8% of mastectomies (71/113), patient-only requests for 27.4% (31/113), both surgeon and patient requests for 4.4% (5/113), and neither documented for 5.3% (6/113) (Table S3.). Surgeon-only requests were significantly more common than patient-only requests (*p* < 0.001).

Agreement between imaging and pathology extent classifications was low across modalities. Cohen’s *κ* was 0.241 for MRI and 0.123 for mammography, indicating only slight agreement beyond chance. Sensitivity analyses stratified by imaging source and neoadjuvant treatment status yielded no meaningful changes in the magnitude or direction of the primary associations.

## Discussion

This study demonstrates a substantial rate of imaging–pathology discordance among patients undergoing mastectomy, with nearly half of cases (47.9%) meeting criteria for discordance-driven mastectomy rate (DDMR), defined as imaging overestimation (imaging-extensive disease with localized final pathology). Importantly, imaging-defined extent alone may not reliably predict final pathologic burden, particularly in tumors with non-mass enhancement or DCIS components. Imaging overestimation was substantially more common than underestimation, consistent with the known sensitivity and specificity characteristics of breast MRI and mammography. The predominance of overestimation suggests that imaging-defined extent may influence surgical planning in this population.

Prior studies have demonstrated increased mastectomy utilization associated with preoperative MRI without consistent improvements in surgical or oncologic outcomes.^1,11,15,16^ Our findings suggest that imaging–pathology discordance, particularly overestimation of disease extent, may contribute to this pattern.

The overestimation patterns observed across histologic subtypes are consistent with prior findings demonstrating that MRI frequently overestimates tumor extent. Onesti et al. reported MRI overestimation in approximately one-third of cases, often related to suspected satellite lesions or surrounding DCIS.^9^ In the present study, overestimation was most pronounced in mixed histology and DCIS-containing tumors, supporting prior observations that non-mass enhancement and diffuse in situ components are particularly prone to radiologic exaggeration. Similarly, Mennella et al. identified DCIS histology as a major factor associated with MRI–pathology discordance.^17^ Both MRI and mammography demonstrated low agreement with final pathology and a consistent tendency toward overestimation. Overestimation patterns also varied by imaging modality, with the highest rates observed in cases with mammography-defined extent-indicating features, followed by MRI and combined modality findings. These findings reinforce the limitations of relying on preoperative imaging alone to define disease extent and support integrating imaging findings with clinical and pathologic judgment during surgical planning.

Non-mass enhancement appears to play an important role in imaging–pathology discordance. In the present study, discordance-driven mastectomy occurred only among patients with at least one imaging modality demonstrating features suggesting extensive disease, whereas no DDMR events were observed among patients without such findings on either MRI or mammography. Imaging features suggesting extensive disease, however, did not reliably distinguish true extensive disease from imaging overestimation. Prior studies support this observation. Kwon et al. identified non-mass enhancement and multicentric lesions on pre-neoadjuvant MRI as independent predictors of radiologic–pathologic discordance.^18^ Similarly, Rominger et al. found that non-mass enhancement lesions were substantially more likely to be overestimated than mass lesions.^19^ Together, these findings suggest that specific imaging characteristics, particularly non-mass enhancement, may contribute to discordance in clinical practice.

Neoadjuvant chemotherapy (NACT) introduces additional complexity in interpreting imaging–pathology discordance. Although systemic therapy may improve concordance by reducing tumor burden, prior studies have shown that radiologic–pathologic discordance on post-treatment imaging remains common, even among patients with substantial treatment response. Kwon et al. reported that posttreatment MRI findings, particularly non-mass enhancement and multicentric patterns, were independently associated with radiologic–pathologic discordance following NACT.^18^ In the present study, imaging classification in patients receiving NACT was based on the most recent preoperative imaging prior to surgery, reflecting the imaging used for final surgical planning. However, posttreatment imaging findings may represent fibrosis, treatment effect, or residual enhancement rather than viable tumor. As a result, cases classified as imaging overestimation in this subgroup may not reflect true misclassification of baseline disease extent. Consistent with prior work, discordance-driven mastectomy rates were similar between patients undergoing upfront surgery and those receiving NACT, although these findings should be interpreted cautiously given the known limitations of post-treatment imaging assessment. Future studies analyzing NACT-treated patients separately may further clarify the relationship between imaging-defined extent and final pathology.

Prior work by Katz et al. demonstrated that both surgeon recommendations and patient involvement significantly influence mastectomy decisions, with greater patient involvement associated with higher mastectomy rates when multiple surgical options are considered appropriate.^20^ In addition, Lee et al. identified substantial patient knowledge gaps regarding local recurrence risk and the survival equivalence of mastectomy and breast-conserving surgery.^21^ In the present study, both surgeons and patients contributed to discordance-associated mastectomy cases, however, surgeon-driven decisions were more common than patient-driven decisions suggesting that when imaging suggests extensive disease, these concerns may become amplified and generate a larger contribution of surgeon-driven decisions of more extensive surgery even when final pathology ultimately supports localized disease.

These findings have important implications for surgical planning. The key issue is not whether imaging identifies additional abnormalities, but whether those findings accurately reflect true pathologic extent. In this cohort, imaging features suggesting extensive disease were frequently associated with localized final pathology supporting the consideration of additional confirmatory strategies—such as targeted biopsy, multidisciplinary radiologic–pathologic review, or interval reassessment—before recommending mastectomy in patients who may otherwise remain candidates for breast-conserving surgery.^22^

Additionally, these observations may help inform preoperative counseling and multidisciplinary decision-making. Surgeons should discuss the known limitations and potential overestimation patterns of breast imaging during preoperative counseling. Multidisciplinary radiologic–pathologic review may help better contextualize imaging abnormalities before recommending mastectomy. In selected cases, additional image-guided biopsies or interval reassessment may further improve radiology–pathology concordance and support surgical decision-making.^23^

Nevertheless, imaging–pathology discordance should not be interpreted as synonymous with inappropriate surgical management. Decisions regarding mastectomy are influenced by multiple factors not captured in this analysis, including genetic risk, family history, breast-to-tumor ratio, reconstructive considerations, patient preference, and concerns regarding radiation therapy. In addition, clinically suspected multifocality may not be fully represented within the binary pathologic classification used in this study. Therefore, DDMR should be interpreted as a measure of discordance rather than a direct measure of avoidable surgery.

Several limitations should be acknowledged. The retrospective design limits causal inference and may not fully capture the complexity of surgeon–patient decision-making. In addition, the binary classification of imaging and pathology findings used in this study simplifies a continuous spectrum of disease extent and may not fully reflect real-world surgical decision-making. While the > 5 cm threshold and/or multifocality/multicentricity criteria provided a practical framework for analysis, in clinical practice, eligibility for breast-conserving surgery depends on additional factors beyond tumor size, including tumor distribution, breast anatomy, and anticipated cosmetic outcomes. As a result, categorizing disease extent as simply localized or extensive may oversimplify a more complex clinical decision process. Future studies using continuous tumor size measurements or separate analyses of size and multifocality may further refine understanding of imaging–pathology discordance.

Because this study included only patients undergoing mastectomy, it cannot determine whether imaging findings directly influenced conversion from breast-conserving surgery to mastectomy. The single-center design may limit generalizability, as imaging interpretation and surgical practices vary across institutions. Imaging abstraction was performed using standardized predefined criteria; however, interrater reliability was not formally assessed. Assessment of decision drivers relied on chart documentation and may not fully reflect clinical discussions or patient motivations. Patient-level demographic variables, including race, ethnicity, socioeconomic status, and education level, were not consistently available and therefore were not included in the analysis, although these factors may influence both patient preferences and clinician recommendations. Long-term oncologic outcomes were not evaluated, limiting assessment of whether discordance-associated mastectomies affected recurrence or survival outcomes. Finally, some subgroup analyses were limited by small sample sizes, and surgeon- and radiologist-level variability were not assessed.

## Conclusions

This study demonstrates that imaging–pathology discordance characterized by overestimation of disease extent is common among patients undergoing mastectomy. Poor agreement between imaging and final pathology across modalities highlights the limitations of relying on imaging-defined extent alone in surgical planning. These findings support careful multidisciplinary interpretation of imaging findings, improved patient counseling regarding imaging uncertainty, and consideration of confirmatory strategies before irreversible surgical escalation.

## Supplementary Information

Below is the link to the electronic supplementary material.

Supplementary file1 (DOCX 16 KB)
