# Supplementary material for: Preoperative Overestimation of Disease Extent and Discordance-Driven Mastectomy: A Retrospective Review of 245 Cases
Source: Ann Surg Oncol. 2026 Jul 17;33(11):9761–8. doi: 10.1245/s10434-026-20137-x (PMC13630832; doi:10.1245/s10434-026-20137-x)
Supplement: Supplementary file 1 [file 10434_2026_20137_MOESM1_ESM.docx]

**Supplementary section tables:**

**Table S1. Imaging overestimation rates within histologic subtypes**

Rates represent the proportion of patients within each histologic subtype in whom preoperative imaging overestimated disease extent relative to final pathology. Values are presented as number overestimated per total in subtype (k/n), percentage, and 95% confidence interval.

| **Histologic subtype** | **Overestimated (k)** | **Total (n)** | **Overestimation rate (%)** | **95% CI** |
| --- | --- | --- | --- | --- |
| DCIS-containing | 21 | 44 | 47.7 | 32.7–63.1 |
| Invasive ductal carcinoma (IDC) | 48 | 108 | 44.4 | 35.0–54.3 |
| Invasive lobular carcinoma (ILC) | 8 | 24 | 33.3 | 16.4–55.3 |
| Mixed histology | 34 | 56 | 60.7 | 46.8–73.2 |
| Other | 2 | 4 | 50.0 | 15.0–85.0 |

**Table S2. Imaging Overestimation (Discordance-Driven Mastectomy) Stratified by Neoadjuvant Chemotherapy Status**

| **NACT** | **N total** | **DDMR Events (%)** | **95% CI** |
| --- | --- | --- | --- |
| No | 151 | 72 (47.7%) | 39.5–56.0 |
| Yes | 85 | 41 (48.2%) | 37.3–59.3 |

**Table S3. Attribution of Mastectomy Requests Among Discordance-Driven Mastectomy (DDMR) Cases**

| **Request Pattern** | **n (k)** | **Total (N)** | **%** |
| --- | --- | --- | --- |
| Surgeon-only | 71 | 113 | 62.8% |
| Patient-only | 31 | 113 | 27.4% |
| Both | 5 | 113 | 4.4% |
| Neither | 6 | 113 | 5.3% |
